# Successful Endoscopic Management of a Renal Fungal Ball using Flexible Ureterorenoscopy

**DOI:** 10.1155/2019/9241928

**Published:** 2019-12-17

**Authors:** Mahmoud Abuelnaga, Sadegh Khoshzaban, Mahmoud Reda Badr, Aasem Chaudry

**Affiliations:** ^1^Department of Urology, Bedford Hospital NHS Trust, Bedford, UK; ^2^Department of Urology, Ain Shams University Hospitals, Cairo, Egypt

## Abstract

**Background:**

Candida as a cause for urinary tract infection in healthy individuals is unusual. The extension of fungi into the urinary collecting system rarely leads to formation of bezoars or fungus balls. This can in turn lead to hydronephrosis, obstructive uropathy and sepsis.

**Case Presentation:**

An eighty years old gentleman presented to A&E with confusion, severe urosepsis and acute kidney injury. CTKUB demonstrated significant right sided hydronephrosis, perinephric fat stranding and gas in collecting system. A year prior to this admission he has become known to the urology team for a fungal ball that was noted in the upper pole of the right kidney which was picked up following elective flexible ureterorenoscopy for right kidney stones. Flexible ureterorenoscopy and successful retrieval of fungal ball by basket was performed.

**Conclusion:**

We advocate this technique to be considered as an alternative to the current treatment offered to patients with fungal ball infections and especially so in cases were a nephrostomy is either contraindicated, unavailable, or not possible.

## 1. Introduction and Background

Candida as a cause for urinary tract infection in healthy individuals is unusual. The fungal species has two ways invading the urinary tract system, either via haematogenous dissemination or via the bladder and urethra referred to as antegrade and retrograde respectively [1]. Kidneys are the second major target organ following the lungs for antegrade invasion by Candida [2]. The extension of fungi into the urinary collecting system rarely leads to formation of bezoars or fungus balls. This can in turn lead to hydronephrosis, obstructive uropathy and sepsis [3].

Given the two possible routes of fungal infection in urinary tract, it can be concluded that the candida infection in the genitourinary tract maybe the cause of systemic infection or as a result of it. Following an extensive review of the cases of urinary tract infections caused by candida albicans, Fisher and associates demonstrated the following as the predisposing factors: diabetes mellitus, antibiotic administration, steroid therapy, urine flow turbulence, congenital anomalies, neurogenic bladder, indwelling catheter, and ileal conduits [4].

Fungal balls in the pelvis of kidney, also known as fungal accretions or bezoars are known to cause urinary tract obstruction. The diagnosis is often established by presence of fungi in urine culture and identification of obstructive uropathy via imaging studies such as ultrasound, intravenous pyelography and CT scan. These imaging could also identify soft tissue densities within the renal collecting system, pelvis or ureter [5].

This case report aims to demonstrate an alternative method for management of candiduria due to fungal ball in the kidney. Most reported cases in literature were managed by intravenous antifungal treatment and antifungal instillation through percutaneous nephrostomy tube [6].

## 2. Case Presentation

An eighty-year-old patient,who was later found to have a right sided fungal ball causing obstructive uropathy, initially presented in 2017 with renal colic. He was known to have uncontrolled type two diabetes mellitus and a history of neo-adjuvant long course chemo-radiotherapy followed by low anterior resection and loop ileostomy for management of a rectal tumour.

At the time of initial presentation, he was found to have a left sided 1.2 cm mid ureter calculus for which he had a left rigid ureteroscopy and stent insertion. A follow-up ultrasound scan of his kidneys revealed bilateral small kidney stones and he subsequently had shockwave lithotripsy on the left side.

During this time the patient was admitted to the hospital under the medical team for management of diabetic ketoacidosis and it was noted that his urine culture grew candida. He came under our attention two months later when he was booked for an elective flexible ureterorenoscopy for management of his right sided kidney stones. However it was noted on URS that he had a fungal ball in the upper pole of the right kidney and therefore a JJ stent was inserted and IV antifungals were started as per microbiologists' recommendation. His creatinine at that time was 3.6 mg/dl.

One month later, he had a right flexible ureteroscopy and laser stone disintegration with partial removal of fungal ball and insertion of JJ stent. His renal function improved following the procedure (Cr 2.2 mg/dl). Since the entire fungal ball could not be removed at the time, a second-look flexible ureteroscopy was planned two weeks later while the patient was sent home with IV antifungals. Unfortunately, the patient declined the procedure following discharge and did not attend follow up clinic appointments.

**Figure 1:**
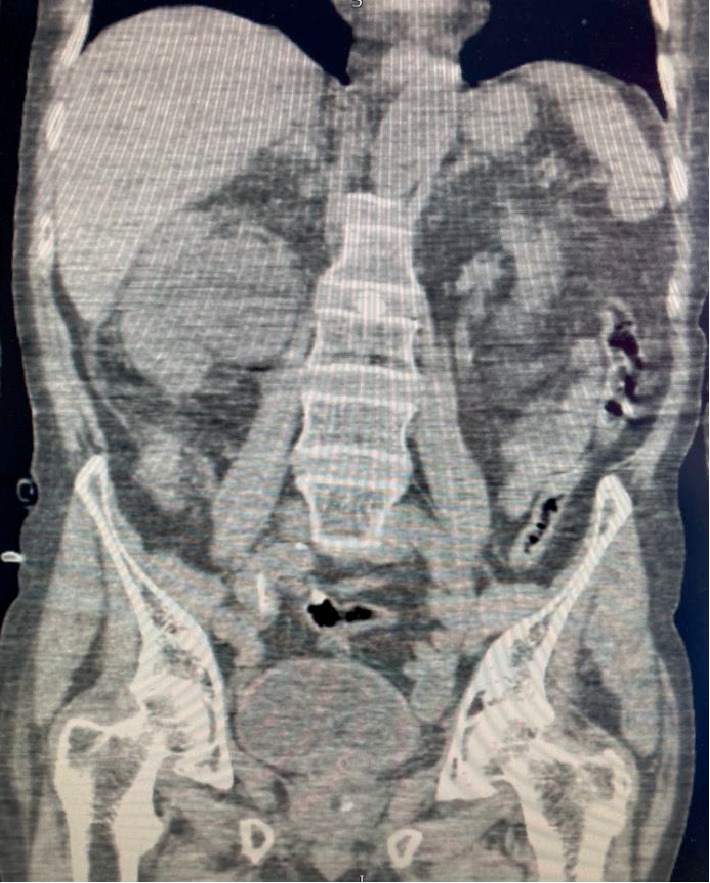
CT KUB showing significant right sided hydronephrosis along with perinephric fat stranding.

Ten months later, he was admitted with severe urosepsis, confusion and acute kidney failure (Cr 4.8 mg/dl). CT KUB showed significant right sided hydronephrosis along with perinephric fat stranding and gas in the collecting system (Figure 1).

**Figure 2:**
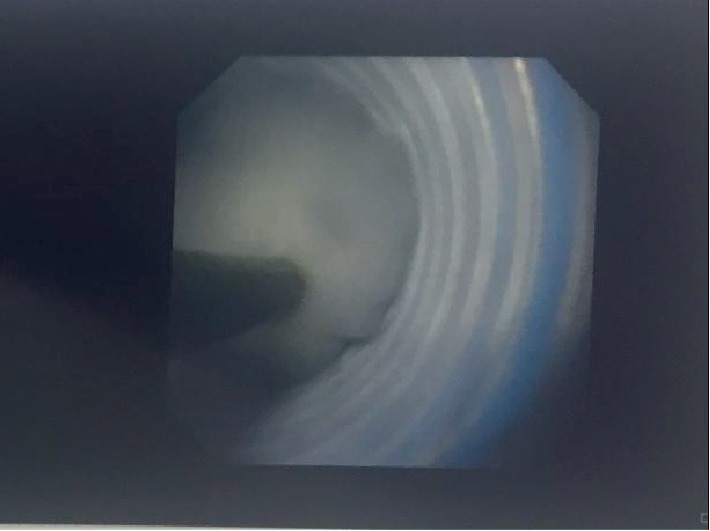
Fungal tissue inside basket within the ureteral access sheath.

**Figure 3:**
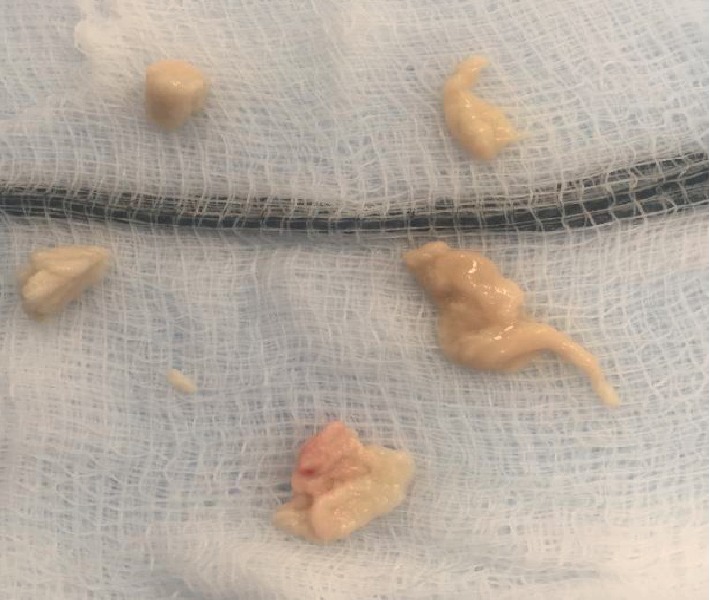
Retrieved fluffy fungal tissues by basket.

The patient underwent an urgent cystoscopy and replacement of blocked JJ stent initially following which the patient's general condition as well as renal function improved (Cr 2.2 mg/dl). During the same admission he subsequently had a right sided flexible ureteroscopy which went as follows. After the placement of ureteral access sheath, 200 mls of pure pus was drained and sent for culture. A Zero Tip Nitinol Stone Retrieval Basket was then used to remove the fluffy fungal tissues and a JJ stent was left in place (Figures 2 and 3).

The culture heavily grew candida albicans and reported as sensitive to Fluconazole. Analysis of the tissue retrieved also showed granular debris together with numerous fungal spores and hyphae which were consistent with Candida species. He was then discussed with microbiologist and specialised infectious diseases who advised long term Intravenous Fluconazole for two weeks after complete removal of fungal ball. Two weeks later, he underwent another flexible ureteroscopy and his kidney was found to be clear of fungal tissues and his stent was removed. His condition was stable, and his creatinine level settled at 1.9 mg/dl when he was discharged home.

## 3. Discussion

Mycosis renalis, (fungal infection of kidneys) as described by Lundquist in 1931 is a rare and serious infection which is even less common as a primary infection. Lundquist first reported on 17 patients with primary infections and demonstrated that these infections are even more serious in immunocompromised patients [7].

Common predisposing factors of fungal infections in the urinary tract are diabetes mellitus, prolonged Foley catheter, urinary tract abnormalities, prolonged antibiotic therapy, steroids, immunosuppressive therapy, malnutrition and malignancy. Formation of a fungal ball can be initiated by agglutination of a necrotic tissue nucleus (papillary necrosis), mucosus debris and foreign or stone debris. Such a fungal ball can in turn cause upper urinary tract obstruction with hydronephrosis . Most frequent pathogens seen to cause such complications are *C. albicans* and *C. tropicalis* [3, 8].

In complex fungal infections of the urinary tract, upper urinary tract imaging is essential due to high incidence of obstructive uropathy in complex fungal infections. Wainstein reviewed fifty patients who had fungal infections of the urinary tract from 1982 to 1992 and classified the fungal infections into simple infections (i.e., localised to bladder) and complex infections (i.e., those involving upper urinary tract or with systemic infections) and demonstrated that the incidence of the following were significantly higher in patients with complex infections. These, in descending order of difference in incidence, were obstructive uropathy, malnutrition, neoplasia, prolonged antibiotic use and renal failure. The high incidence of obstructive uropathy in complex infection (88%) together with the fact that fungemia being common in complex infections (81%) with a 36% mortality rate, makes upper urinary tract imaging a requirement in such patients [8].

Most of the reported cases of fungal ball in literature were managed by either long term antifungal therapy or local antifungal instillation through a nephrostomy tube [2, 3, 6, 9, 10]. Current management options for renal fungal ball include intravenous antifungal agents, percutaneous nephrostomy with instillation of antifungal agents with no consensus over the duration of instillation required. The most common antifungal agents used are fluconazole and amphotericin B due to their high urine concentration, however, amphotericin B is also nephrotoxic which complicates its use in such scenarios. Other antifungals, such as echinocandins, are not renally excreted and therefore their systemic use in such conditions is limited [11, 12].

There are very limited number of cases in the literature about endoscopic removal of fungal ball [13] and prior to the advent of endourology, open surgical procedures with pyelotomy were sometimes necessary for removal of fungal balls in the renal collecting system. The current mainstay of treatment is percutaneous nephrostomy which is used to successfully manage patients with obstructive uropathy secondary to such infections. Nephrostomy both relieves the obstruction and also provide direct access for antifungal instillation as well as percutaneous and identification and possible removal of fungal ball [14, 15].

In current practice, endoscopy and flexible ureterorenoscopy is widely available in most hospitals across UK. The use of such equipment can be helpful in management of patient in fungal ball as it can both expedite treatment and reduce length of hospital stay for such patients. This is in direct contrast with the variable and often prolonged treatment that is required with the use of current established treatment with nephrostomy and direct antifungal instillation which averages around seven days [1].

Nephrostomy service can be limited in many hospitals and is not as widely available as urological endoscopy services across UK. Furthermore, nephrostomy is contraindicated in patients with bleeding disorders and uncooperative patients. Musculoskeletal deformities, body habitus and not adequately dilated renal pelvis can also complicate nephrostomy placement [6].

## 5. Conclusion

Herein we demonstrated that endoscopic retrieval of renal fungal tissues using flexible ureterorenoscopy is an effective, safe and rapid intervention in dealing with an obstructing renal fungal ball. It can also significantly reduces hospital stay and toxicity associated with long-term use of either local or systemic antifungals .We advocate that it should be considered as an alternative to the current treatment offered to patients with fungal ball infections and especially so in cases were a nephrostomy is either contraindicated, unavailable, or not possible.
